# In vivo immobilization of an organophosphorus hydrolyzing enzyme on bacterial polyhydroxyalkanoate nano-granules

**DOI:** 10.1186/s12934-019-1201-2

**Published:** 2019-10-10

**Authors:** Ru Li, Jian Yang, Yunzhu Xiao, Lijuan Long

**Affiliations:** 1 CAS Key Laboratory of Tropical Marine Bio-Resources and Ecology, RNAM Center for Marine Microbiology, Guangdong Key Laboratory of Marine Materia Medica, South China Sea Institute of Oceanology, Chinese Academy of Sciences, Guangzhou, 510301 People’s Republic of China; 2 University of the Chinese Academy of Sciences, Beijing, 100049 People’s Republic of China; 3 Shenzhen Key Laboratory of Microbial Genetic Engineering, College of Life Sciences and Oceanology, Shenzhen University, Shenzhen, 518055 Guangdong People’s Republic of China

**Keywords:** Polyhydroxyalkanoates display, Organophosphorus anhydride hydrolase, Bioremediation, Nano-biocatalysts

## Abstract

**Background:**

Polyhydroxyalkanoate (PHA) are nano-granules naturally produced by bacteria. Two types of proteins, PHA synthase (PhaC) and phasins (PhaPs), are attached to the PHA surface by covalent and hydrophobic interactions. Utilizing these anchored proteins, functionalized PHA nano-granules displaying proteins of interest can be easily prepared by fermentation.

**Results:**

In this study, a one-step fabrication method was developed for stable and efficient immobilization of an organophosphorus degrading enzyme on PHA nano-granules. The nano-biocatalysts were produced in recombinant *Escherichia coli* cells into which the polyhydroxyalkanoate synthesis pathway from *Cupriavidus necator* had been introduced. Two different strategies, covalent attachment and hydrophobic binding, were investigated by fusing bacterial organophosphorus anhydride hydrolase (OPAA4301) with PhaC and PhaP, respectively. Using both methods, the tetrameric enzyme successfully self-assembled and was displayed on the PHA surface. The display density of the target fused enzyme was enhanced to 6.8% of total protein on decorated PHA by combination of covalent and non-covalent binding modes. Immobilization of the enzyme on PHA granules resulted in higher catalytic efficiency, increased stability and excellent reusability. The *k*_cat_ values of the immobilized enzymes increased by threefold compared to that of the free enzyme. The pH stability under acidic conditions was significantly enhanced, and the immobilized enzyme was stable at pH 3.0–11.0. Furthermore, more than 80% of the initial enzyme activity retained after recycling ten times.

**Conclusions:**

This study provides a promising approach for cost-efficient in vivo immobilization of a tetrameric organophosphorus degrading enzyme. The immobilization process expands the utility of the enzyme, and may inspire further commercial developments of PHA nano-biocatalysts. As revealed by our results, combination of covalent and non-covalent binding is recommended for display of enzymes on PHA granules.

## Introduction

Many microorganisms naturally produce nanometer scale particles. These biologically produced nanoparticles (BNPs) exist in a wide range of forms including polyhydroxyalkanoates, endospores, exosomes, and magnetosomes [1]. BNPs are composed of a spherical core–shell structure, where the organic or inorganic cores are associated with a number of proteins on their surfaces [2–4]. These surface proteins have attracted much attention and inspired investigations to explore them as anchoring tags to attach proteins and peptides to the surface of BNPs. The engineered BNPs are exciting prospects for drug targeting [5], bio-catalysis [6], biochemical separation [7] and bioremediation [8]. Compared with existing chemical immobilization technologies [9, 10], surface engineering of BNPs is highly advantageous. The functional BNPs usually assemble under mild physiological conditions in a single step, avoiding the requirement for additional external agents. Accordingly, display of proteins on nanoparticles in microbial cells is considered as an emerging alternative to traditional chemical immobilization.

**Fig. 1:**
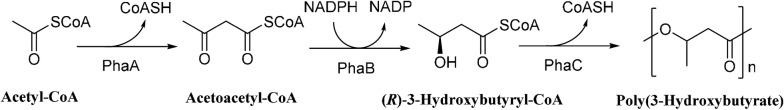
Poly(3-Hydroxybutyrate) biosynthesis catalyzed by PhaA, PhaB, and PhaC

Polyhydroxyalkanoate granules are naturally occurring inclusions used as intracellular carbon and energy storage by many bacteria under conditions of nutrient limitation [11]. The PHA biosynthetic pathway contains three key enzymes, PHA synthase (PhaC), β-ketothiolase (PhaA) and acetoacetyl-CoA reductase (PhaB), composing the PhaABC operon (Fig. 1). Heterogeneous organisms co-expressing these three enzymes have been shown to produce PHAs [12, 13]. The catalytic cysteine residue of PhaC is covalently attached to the growing PHA [14]. This covalent bonding feature of PhaC has commonly been developed as an “anchoring rope” to the surface of PHA granules. Rational engineering of PhaC enables the active display of proteins having a variety of functionalities. In addition to PhaC, non-catalytic small phasin proteins (PhaP1–PhaP7), which have been proposed to regulate the size of PHAs, also associate with PHA granules via hydrophobic interactions [15]. PhaPs are abundant on PHA granules, and thus they have been utilized as affinity tags for effective protein purification [16, 17].

Organophosphorus compounds (OPs) are widely used as pesticides, flame retardants, or plasticizers, which easily cause poisoning to unintended organisms and are recognized as typical organic pollutants [18]. As innovative tools, OPs degrading enzymes have been exploited to detoxify these compounds for environmental remediation, biosensors and therapy [19]. Investigations attempted to expand the application scope of OPs degrading enzymes have attracted a great deal of attention in recent years. For instance, engineered carboxylesterase and lactonase with improved organophosphorus hydrolase catalysis were developed as electrochemical biosensor and decontamination formulation, respectively [20]. High cell density fed-batch strategy was applied to enhance the yield of archaea thermophilic phosphotriesterase [21]. We previously reported a marine bacterial organophosphorus hydrolase OPAA4301 with efficient decontaminating abilities on pesticides such as paraoxon, dichlorvos, and profenofos [22]. In this study, we explore the potential to display the enzyme, OPAA4301 on PHA granules using PhaC and PhaP as anchoring proteins. We hypothesized that: (1) the multimeric enzyme could self-assemble on the surface of the PHA particle; (2) the engineered enzyme fused with PhaC and PhaP could coexist on the PHA particle, and the combination of covalent and non-covalent binding would increase the display level; (3) the displaying PHA nano- granules prepared in one step could be easily harvested and recycled; (4) immobilization could stabilize the enzyme. Functional display on PHA nano- granules was achieved by metabolic engineering in *Escherichia coli* cells. Biochemical characteristics of the immobilized enzymes, including catalytic efficiency, stability and reusability, were systematically investigated.

## Methods

### Bacterial strains, plasmids and reagents

*Escherichia coli* strains T1 (Transgen Biotech, Beijing, China) and BL21 (DE3) (Novagen, Madison, USA) were used for plasmid construction and production of PHA granules, respectively. The pACYCDuet-1 and pETDuet-1 vectors (Novagen), both with two sets of T7 promoter, were used for co-expression of target genes. These two plasmids have different replicons, thus can exist in one *E. coli* cell. The gene encoding organophosphorus hydrolase was amplified from the previously constructed pET-*opaa*4301 plasmid [22]. The PHA granule synthesis genes, *pha*C, *pha*A, *pha*B, and *pha*P, were amplified from *Cupriavidus necator* ATCC 17699, the most investigated poly(3-hydroxybutyrate) producer [23]. The oligonucleotide primers used in this study are listed in Additional file 1: Table S1. Gene manipulation reagents, including T4 DNA ligase, restriction endonuclease, Pfu polymerase, and seamless assembly kit, were all purchased from Transgen Biotech.

### Plasmid construction

**Fig. 2:**
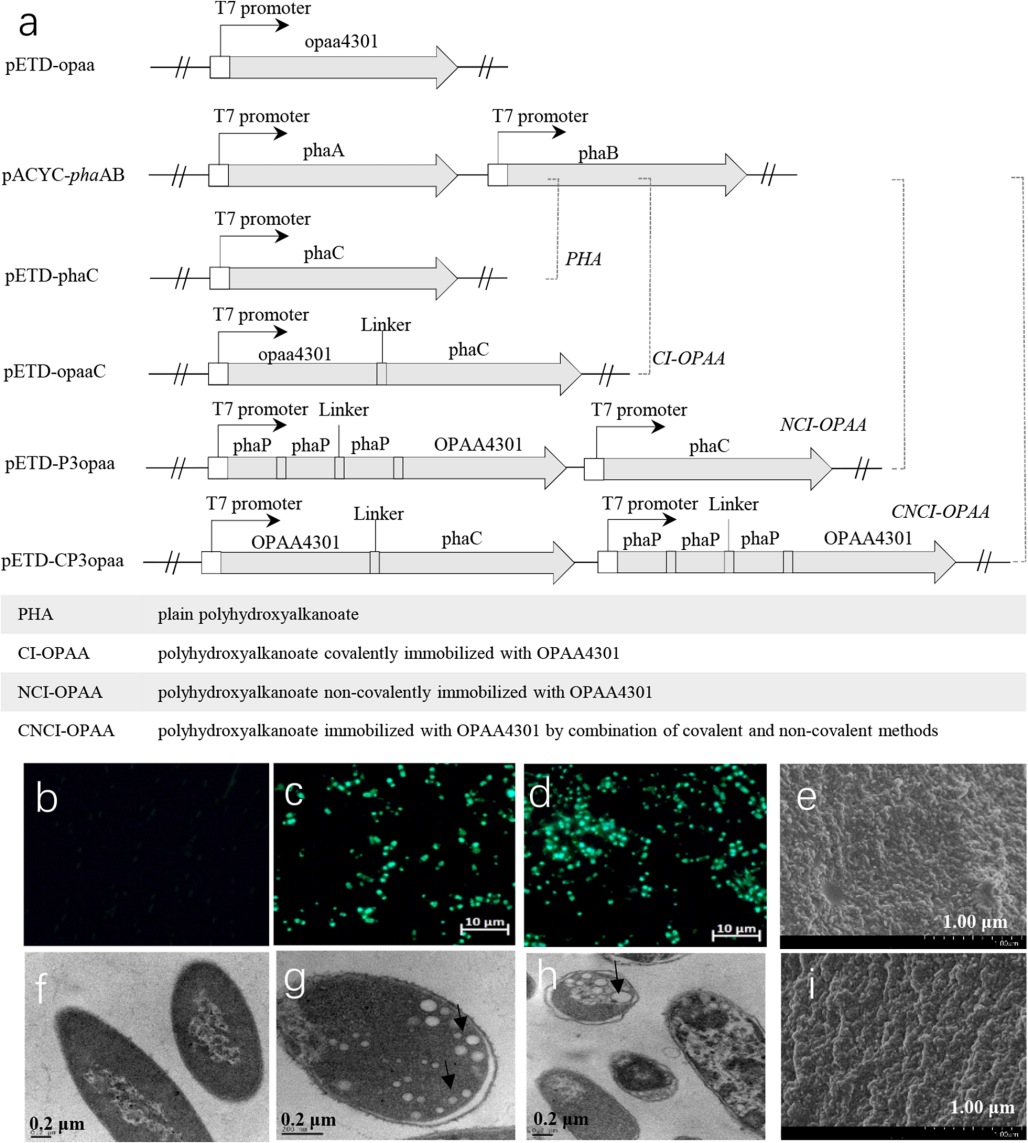
PHA-based nano-biocatalyst formation by co-expression of target enzyme (OPAA4301) and anchored proteins (PhaC and PhaP). **a** Schematic of plasmid construction for metabolic engineering and PHA surface engineering. **b** Fluorescence microscopy analysis of control recombinant *E. coli* carrying pACYCDuet-1 and pETDuet-opaa after staining with Nile blue. **c** Fluorescence microscopy analysis of *E. coli* carrying pACYC-phaAB and pETD-*pha*C indicates formation of PHA in the cells after induction. **d** Fluorescence microscopy analysis of *E. coli* carrying pACYC-phaAB and pETD-CP3opaa indicates formation of engineered PHA in the cells after induction. **e** Scanning electron microscopy analysis of purified PHA. **f** Transmission electron microscopy analysis of control recombinant *E. coli* cells carrying pACYCDuet-1 and pETDuet-opaa plasmids. **g** Transmission electron microscopy analysis of *E. coli* carrying pACYC-phaAB and pETD-*pha*C after induction. PHA granules are indicated by arrows. **h** Transmission electron microscopy analysis of *E. coli* carrying pACYC-phaAB and pETD-CP3opaa. PHA granules with diameters of 20–200 nm were observed. **i** Scanning electron microscopy analysis of purified CI-PHA

The *pha*A and *pha*B fragments amplified from the genomic DNA of *C. necator* ATCC 17699 were digested with two pairs of restriction enzymes (*Nco* I/*Not* I and *Nde* I/*Kpn* I), and inserted under each T7 promoter of the pACYCDuet-1 vector respectively, generating pACYC-phaAB plasmid. The pACYC-phaAB plasmid was responsible for biosynthesis of hydroxyalkanoate. Four pETDuet-1 derivative plasmids, pETD-phaC, pETD-opaaC, pETD-P3opaa, and pETD-CP3opaa, were constructed for the expression of modified *pha*C and *pha*P1 genes. To obtain the fused organophosphorus hydrolase (OPAA4301) and PHA synthase (PhaC) with a linker peptide (GGGSGGGSGGGS), overlap PCR was performed using PhaC-L1/phaC-stop-Not I and opaa4301-no-start-Nco I/phaC-stop-Not I as primers. The resulting hybrid DNA fragment was ligated into the pETDuet-1 plasmid, generating pETD-opaaC plasmid. The pETD-P3opaa plasmid was constructed by inserting *opaa*4301 with *pha*P fused at its 5′-terminus and *pha*C genes under each T7 promoter of pETDuet-1. The *pha*P genes were first amplified from strain ATCC 17699 with three pairs of primers (phaP-Nde I/phaP-L2-Bgl II, phaP-Bgl II/phaP-L2-Kpn I, and phaP-Kpn I/phaP-L2). Each pair of primers contained a linker peptide (NNNNNLGIEGRIS) encoding gene. To generate pETD-CP3opaa, the *pha*C gene of pETD-P3opaa was substituted with the fusion gene of *opaa*4301 and *pha*C subcloned from the pETD-opaaC plasmid. A schematic diagram of the plasmid construction is presented in Fig. 2a. The maps of the whole expression vectors are also shown in Additional file 1: Figure S1. All of the constructed plasmids were confirmed by DNA Sanger sequencing.

### Production and purification of PHA granules

To produce different types of PHA granules, the pACYC-phaAB plasmid was transformed into *E. coli* BL21 (DE3) with pETD-phaC, pETD-opaaC, pETD-P3opaa, or pETD-CP3opaa, respectively. The recombinant *E. coli* BL21 (DE3) cells were cultured in terrific broth (TB) medium (12 g trptone, 24 g yeast extract, 5 g glycerol, 2.3 g KH_2_PO_4_ and 12.5 g K_2_HPO_4_ in 1000 mL water) with ampicillin (50 μg/mL) and chloramphenicol (50 μg/mL) at 37 °C, 200 r/min until the optical density at 600 nm reached 0.6. Isopropyl-β-d-1-thiogalactopyranoside (IPTG) was then added to a final concentration of 1.0 mM to induce expression of heterogeneous proteins. After induction at 30 °C, 200 r/min for 48 h, cells were harvested for the purification of PHA granules as previously described [24]. The induced cells were harvested by centrifugation for 20 min at 4500×*g* and 4 °C. The pellets were washed and suspended in phosphate buffer (50 mM, pH 7.5), and disrupted on ice using a sonicator. The cell lysates (~ 4 mL) were loaded onto a glycerol gradient containing 88% and 44% (v/v) glycerol in phosphate buffer. After ultracentrifugation for 3.0 h at 100,000×*g* and 4 °C, the PHA granules were found in a white layer above the 88% glycerol layer. The PHA granules were withdrawn, washed with 10 volumes of phosphate buffer (50 mM, pH 7.5) and centrifuged at 100,000×*g* for 60 min at 4 °C. The collected granules were then suspended in PBS buffer (1.44 g Na_2_HPO_4_, 0.24 g KH_2_PO_4_, 0.2 g KCl, and 8.00 g NaCl in 1000 mL water) for further analysis. The free enzyme OPAA4301 was prepared as previously described [22].

### Microscopic analysis

The intracellular PHA granules were stained with Nile blue A (Sigma-Aldrich, USA) and visualized by fluorescence microscopy as previously described [25]. Nile blue A (0.25 mg/mL) was dissolved in DMSO and stored in the dark before use. The induced cells were harvested, washed and resuspended in PBS buffer. A sample of cell suspension (1 mL) was stained with 3 μL of Nile blue A solution at room temperature for 30 min. To avoid fluorescence quenching, the whole staining process was conducted under lucifugal conditions. A sample of stained cells (5 μL) was loaded onto a glass slide for fluorescence microscopy analysis (Carl Zeiss, Axio Imager 2) at an excitation wavelength of 460 nm. For transmission electron microscopy analysis, the recombinant cells were washed three times with PBS buffer and fixed with 2.5% glutaraldehyde in the same buffer for 1 h at room temperature. After washing with PBS buffer again, the cells were prepared for transmission electron microscopy as described by Witte et al. [26]. Transmission electron microscopy was performed using a Hitachi Jeol 1230. The PHA granules was extracted according the previous work [27]. Briefly, the PHA granules were extracted by adding 10 times chloroform (v/w) to the lyophilized cells, and incubated at 40 °C for 2 h, then residual biomass was removed via filtration. The granules were precipitated with fivefold volume quantities of cold ethanol (*v*/*v*). The dried PHA granules was observed by scanning electron microscope Hitachi S3400N.

### Analysis and quantification of attached proteins on PHA nano-granules

The total protein associated with purified PHA granules was quantified using the Bradford method [28], and the concentration of fused enzyme was determined by mass spectrometry. Lyophilized PHA granules (1 mg) were digested with 4 μg trypsin (Promega) in 40 μL 25 mM NH_4_HCO_3_ buffer overnight at 37 °C, and the resulting peptides were collected as a filtrate. The peptides from each sample were desalted on C18 Cartridges (Empore™ SPE Cartridges C18, bed i.d. 7 mm, volume 3 mL, Sigma), concentrated by vacuum centrifugation and reconstituted in 40 µL of 0.1% (v/v) formic acid. The peptide content was estimated by UV light spectral density at 280 nm using an extinction coefficient of 1.1 for 0.1% (g/L) solution that was calculated on the basis of the frequency of tryptophan and tyrosine residues in vertebrate proteins. LC–MS/MS analysis was carried out on an Easy nano LC Liquid Chromatograph (Thermo Scientific) which was connected to a Q Exactive Mass Spectrometer (Thermo Scientific). The raw data was processed by MaxQuant 1.3.0.5. A customized database was constructed and used for protein identification and quantification of fused enzyme.

### Enzyme assay

The organophosphorus hydrolase activity of the immobilized enzyme was measured according to the previously described method using paraoxon as substrate [29]. Typically, diluted active PHA granule suspension (50 μL) was added to the substrate solution (250 μL) containing 0.5 mM paraoxon, 0.1 mM MnCl_2_, and 50 mM glycine–NaOH buffer (pH 8.5). The mixture was incubated at 55 °C for 30 min, and then the reaction was stopped by heating at 95 °C for 10 min. The assay was monitored using the absorbance of the *p*-nitrophenol product by spectrophotometry at 405 nm. The PHA granules were used as negative control. The total protein associated with purified PHA granules was quantified using the Bradford method, and The PHA turbid solution was homogeneously dispersed by sonication. One unit of paraoxonase activity was defined as the amount of enzyme that catalyzed the liberation 1.0 μmol of *p*-nitrophenol per minute at 55 °C. To obtain the kinetic parameters of PHA granules and free OPAA4301, six concentration intervals of paraoxon (0.5 to 3.0 mM) were set to monitor the catalytic activities under standard conditions. The *K*_m_ and *k*_cat_ values were calculated according to Michaelis–Menten equation. All the enzyme assays were conducted three times.

### The effect of pH and temperature on activity and stability of immobilized and free OPAA4301

To determine the optimal temperature of the PHA-OPAA4301, the reaction mixture was incubated at various temperatures (30 to 70 °C) in 50 mM glycine–NaOH, pH 8.5 for 1 min prior to activity measurement at the same temperature by addition of substrate. Thermal stability assays were conducted by pre-incubating the purified enzyme at a series of temperature (30–75 °C) for 15 min before determination of the residual activities at 55 °C. The activity of the unheated enzyme was defined as 100%.

The effect of pH on the immobilized enzyme activity was measured by incubating the reaction mixture at pH values ranging from 6.0 to 9.5 for 30 min. In order to investigate pH stability of immobilized and free OPAA4301, the enzymes without MnCl_2_ and substrate were pre-incubated in buffers of different pH values at 4 °C for 2 h. Aliquots were withdrawn to determine the remaining activity under standard condition. Different buffer systems (50 mM) were used: acetic acid-sodium acetate buffer (pH 3.0–6.0), sodium phosphate (pH 6.0–8.0), Tris–HCl (pH 8.0–9.0), glycine–NaOH (pH 8.5–11.0).

### Reusability of enzyme-bearing PHA granules

To assess the reusability of the enzyme-displaying PHA, reaction mixtures were prepared and assayed as described above. At the end of the incubation period, samples were centrifuged at 10,000×*g* for 30 min. The supernatant was removed and analyzed. The PHA granule pellets were then incubated in 300 μL of substrate solution containing 50 mM glycine–NaOH buffer (pH 8.5), 0.5 mM paraoxon, and 0.1 mM MnCl_2_. The cycle was repeated eight times.

## Results

### Display of organophosphorus hydrolase on PHA granules via anchored proteins

To enable production of PHA granules in *E. coli* BL21 (DE3), the hydroxyalkanoate biosynthetic pathway from *C. necator* ATCC 17699 was first introduced to the BL21(DE3) strain using pACYC-phaAB transforming plasmid encoding β-ketothiolase and acetoacetyl-CoA reductase (Fig. 2a). The hydroxyalkanoate monomers were then polymerized to PHA via catalysis by the polyhydroxyalkanoate polymerase, phaC, in vivo. After inducing co-expression of phaA, B, and C genes, PHA granules could be observed by fluorescence microscopy after staining with Nile blue (Fig. 2b–d). The PHA granules tended to accumulate in the poles of recombinant cells (Fig. 2e–g), and the diameters of the granules were in the range of 20–150 nm, and mainly distributed in the range of 40–50 nm (Fig. 2h, i, and Additional file 1: Figure S2).

Since PhaC and PhaP can attach to the surface of PHA granules in different modes, two binding strategies were investigated to generate immobilized enzyme on PHA granules. Covalently immobilized enzyme (CI-OPAA) was produced by fusing the organophosphorus hydrolase OPAA4301 to PhaC at its N-terminus, and non-covalently binding enzyme (NCI-OPAA) was prepared by fusing OPAA4301 to three copies of PhaP1 at the C-terminus. In addition, the combined version of both covalently and non-covalently immobilized enzyme (CNCI-OPAA) was produced in the recombinant *E. coli* harboring the pACYC-phaAB and pETD-CP3opaa vectors (Fig. 2a).

**Fig. 3:**
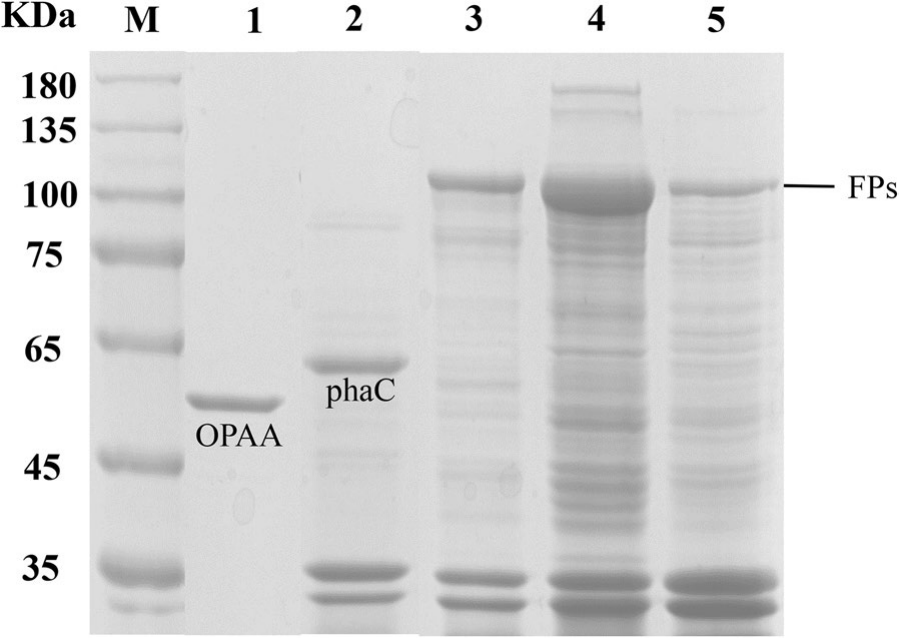
The SDS-PAGE analysis of PHA granule-associated proteins. Lane M, protein molecular weight standard; Lane 1, purified organophosphorus hydrolase enzyme OPAA4301; Lane 2, PHA granules isolated from recombinant *E. coli* harboring pACYC-phaAB and pETD-phaC; Lane 3, PHA granules isolated from recombinant *E. coli* harboring pACYC-phaAB and pETD-opaaC; Lane 4, PHA granules isolated from recombinant *E. coli* harboring pACYC-phaAB and pETD-CP3opaa, Lane 5, PHA granules isolated from recombinant *E. coli* harboring pACYC-phaAB and pETD-P3opaa. (FP, phaC, and OPAA indicate the corresponding bands of fusion proteins, PHA synthetase, and the enzyme OPAA4301, respectively)

The chemical properties of PHA were confirmed by FT-IR (Additional file 1: Figure S3) and NMR (Additional file 1: Figure S4) analysis. SDS-PAGE analysis of the three groups of purified PHA granules clearly showed the formation of fused enzymes on the PHA granules (Fig. 3). Protein bands with an expected molecular weight of 115 kDa were observed for CI-OPAA, NCI-OPAA, and CNCI-OPAA. LC–MS/MS analysis was conducted to identify and quantify the proteins associated with the PHA granules Additional file 2). The results indicated that the target fused proteins were immobilized on the PHA granules, comprising approximately 5% of the total attached protein (Table 1). The ratio of fused protein was higher than 0.985% described previously [6].

**Table 1:** Biochemical characteristics of free and immobilized organophosphorus hydrolase OPAA4301

Enzyme	*T*_opt_ (°C)	pH_opt_	Specific activity (U/mg)	*k*_cat_ (s^−1^)	*K*_m_ (mM)	*k*_cat_/*K*_m_ (M^−1^/s)	Ratio of target enzyme (%)
OPAA4301	55	8.5	1.648 ± 0.0222	3.0 ± 0.526	3.203 ± 0.929	935 ± 89	–
CI-OPAA	55	8.5	0.096 ± 0.0047	11.904 ± 3.893	6.188 ± 2.490	1961 ± 138	4.726
NCI-OPAA	50	8.0	0.109 ± 0.0014	11.223 ± 1.752	6.116 ± 1.299	1850 ± 104	5.606
CNCI-OPAA	55	8.0	0.112 ± 0.0044	–	–	–	6.850

### Paraoxonase activity of immobilized organophosphorus hydrolase

The tetrameric OPAA4301 enzyme self-assembled on the surface of PHA granules was revealed by examining its ability to hydrolyze paraoxon. Each of the engineered PHA granules freeze dried and quantified at a final concentration of 1 μg/mL hydrolyzed 95% of the paraoxon in test samples within 3 h (Additional file 1: Figure S5). The specific activities of CI-OPAA, NCI-OPAA, and CNCI-OPAA towards paraoxon were determined as 0.096, 0.109 and 0.112 U/mg protein, respectively. Kinetic analysis showed that the catalytic activity of the immobilized enzyme was improved. The immobilized CI-OPAA and NCI-OPAA enzymes shared similar *k*_cat_/*K*_m_ values of 1961 ± 138 and 1850 ± 104/M/s, which were twice that of the free OPAA4301 enzyme. The higher catalytic efficiency of the immobilized enzymes was mainly due to increased *k*_cat_ values. The higher *k*_cat_ is likely ascribe to the favorable interaction between the carrier and the enzyme, which made the enzyme fold into optimized conformation on the surface of the PHA granules [30].

### Improved stability of nano-particle enzymes

**Fig. 4:**
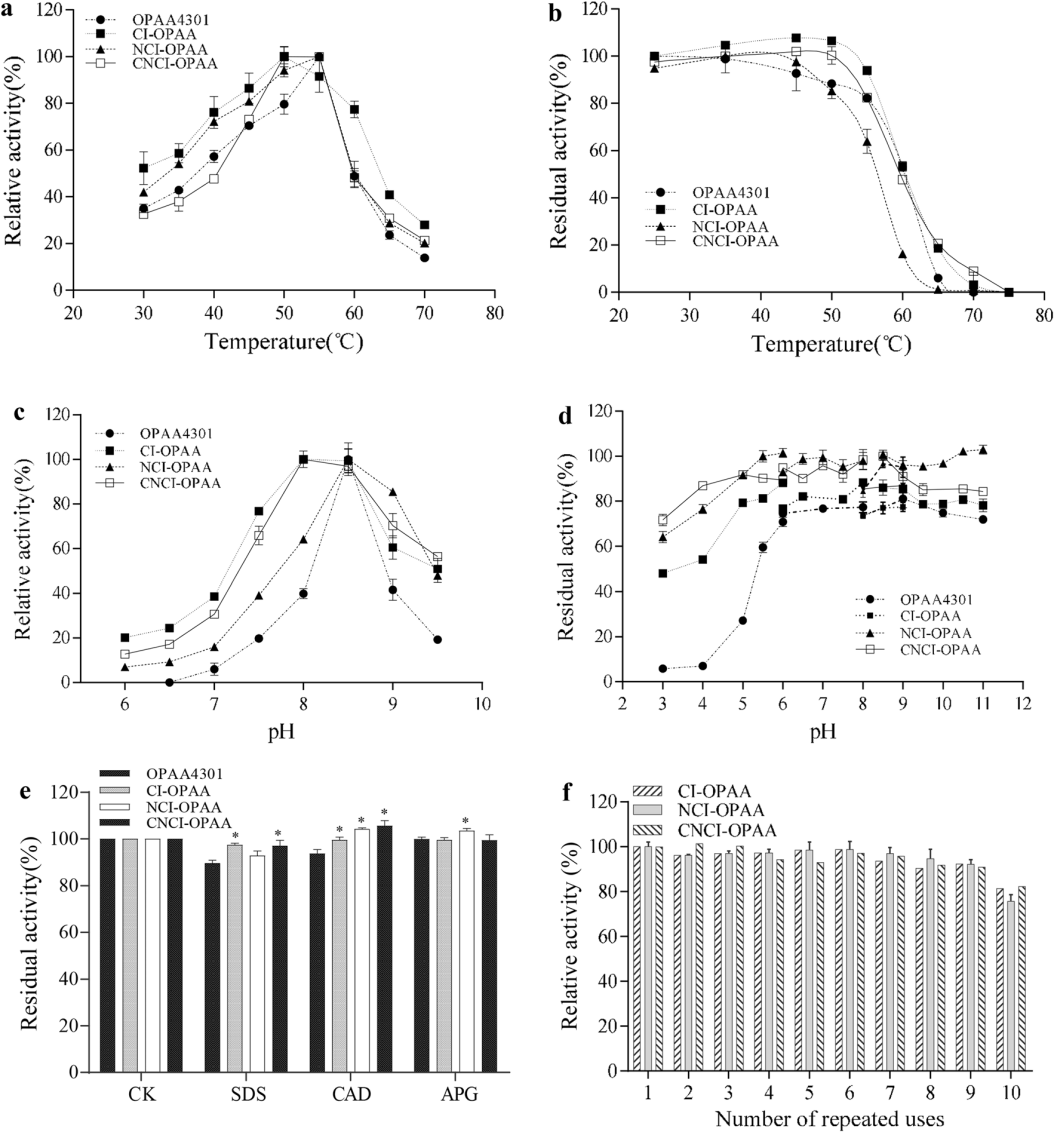
Stability and reusability of PHA immobilized enzymes. **a** Temperature dependency of immobilized enzyme activity. **b** Thermal inactivation profile of PHA immobilized OPAA4301. **c** Effect of pH on the activity of immobilized OPAA4301. **d** pH stability of PHA immobilized enzymes. **e** The effect of detergents on immobilized OPAA4301. The enzymes were pretreated in solution containing 0.1% of different detergents at 4 °C for 30 min before determination of the residual activity. A single star indicates p < 0.05. **f** Reusability of PHA immobilized enzymes. The relative activity was defined as the ratio of activity after each cycle to the initial activity

The effects of temperature on the activity of free and immobilized enzymes was monitored from 30 to 70 °C. The immobilized enzymes shared similar temperature profiles with free OPAA4301 enzyme. All exhibited maximal activity at 55 °C, and were rapidly inactivated at temperatures higher than 60 °C (Fig. 4a). Thermal stability of these enzymes was examined by incubation at different temperatures for 15 min followed by determination of residual activity. The *T*_50_^15^ values (the temperature at which the enzyme retains 50% of the original activity after incubating for 15 min) were 56 and 60 °C for NCI-OPAA and the other three enzymes, respectively (Fig. 4b).

Immobilization of OPAA4301 on PHA granules, as revealed by observations of characteristics at different pH values, can broaden the range of pH adaptation. Even though the optimal pH remained largely unchanged at 8.5, the immobilized enzymes showed higher activities than free enzyme under both acidic and alkaline conditions (Fig. 4c). More interestingly, the pH stability of the enzyme was significantly enhanced. Free OPAA4301 completely lost activity after incubation at pH 3.0, whereas the immobilized enzymes retained more than 50% of their initial activities (Fig. 4d). The obtained property will widen the applicability of immobilized OPAA4301, and makes it superior over free OPAA4301 and other OP hydrolases [31, 32].

### Effects of surfactants and reusability of immobilized enzymes

Three surfactants commercially used in the detergent industry, including anionic surfactant sodium dodecyl sulfate (SDS), non-ionic surfactant alkyl glycosides (APG) and natural surfactant coconut diethanolamide (CAD), were assessed for effects on the stability of immobilized enzymes. As shown in Fig. 4e, all tested surfactants had toxic effects on the free enzyme OPAA4301, while their negative effects on catalysis were clearly eliminated after displaying the enzyme on PHA granules.

Reusability is an important feature of immobilized enzymes that can significantly reduce the costs of bio-catalytic processes. Ten reaction cycles with recovered material were carried out to examine the reusability of the active PHA nano-granules bearing OPAA4301. The results indicated excellent reusability of these immobilized enzymes. More more than 80% of the initial activity still remained after ten cycles for all three immobilized OPAA enzymes (Fig. 4f).

## Discussion

This study demonstrated the functional display of a bacterial tetrameric organophosphorus hydrolase on the surface of PHA granules, which was achieved by co-expression of target enzyme and surface anchoring proteins in *E. coli*. Immobilization of enzymes on natural or synthetic solid supports can increase the robustness of biocatalysts and reduce process costs. Superior to the traditional immobilization technologies of adsorption, embedding, or cross-linking, enzyme display on BNPs can be achieved in a one-step process. Moreover, BNP-based immobilization is carried out under mild physiological condition, which has little negative influence on enzymatic activity. Bacterially produced PHAs, which are biodegradable and biocompatible polyesters, are recognized as excellent carriers for enzymes and drugs. The first reported immobilization of enzyme on PHA involved fusion of β-galactosidase with PhaC [33]. Subsequent studies of immobilized enzymes on PHA granules have generally exploited PhaC to generate covalently anchored active nano-particles [6, 8, 34].

In our study, both covalent and non-covalent binding strategies were investigated and compared for the first time. From our results, non-covalent anchoring mediated by PhaP proteins can result in a greater abundance of associated enzymes on the PHA granules than the PhaC-mediated method. Moreover, unlike previous research on covalent or non-covalent anchoring, the combination of both binding modes is recommended to maximize specific activity and the amount of target enzyme displayed on the engineered PHA surface (Table 1). Nevertheless, the majority of the attached proteins on the engineered PHA are still from *E. coli*. We speculate that by using more other anchoring proteins, such as PhaP2–PhaP7, higher display density can be achieved.

The enzyme OPAA4301 and its homologous enzymes are all reported as dimers or tetramers (dimer of dimers). The dimeric state of the enzyme is theoretically important to the catalysis of the enzyme, since the 45^th^ and 89^th^ residues of one molecule of the enzyme can stretch into the catalytic center to form the substrate binding pocket. Our previous report also confirmed the importance of these two residues on catalysis [22]. We suppose the enzyme self-assembled by the fact that the catalytic ability can be detected. Immobilization of enzyme on solid materials is always associated with changes of protein structure, and hence affects the enzymatic properties. The enzyme kinetics and stability of OPAA4301 were improved after immobilization on PHA, suggesting that display on PHA is a promising strategy to extend the utility of the enzyme. The *k*_cat_ values of CI-OPAA and NCI-OPAA both increased by threefold compared with free OPAA4301 (Table 1). Our results found superior catalytic compared to other PHA displaying organophosphorus hydrolase OpdA, the *k*_cat_ value of which decreased by 16-fold [8]. It is also worth noting that immobilization of OPAA4301 confer stability to acidic and higher temperature conditions (Fig. 4a–d). Our results showed improved enzyme stability, which was similar to that observed in studies on polygalacturonate lyase and α-amylase immobilized on PHA. The polygalacturonate lyase displayed on PHA beads retained its initial activity after incubation at pH buffer from pH 7.0 to 9.0 for 24 h [6]. The α-amylase-bearing particles exhibited better thermal stability and pH tolerance than the free-state enzyme [35]. One possible reason for enhancement on enzyme activity and stability could be attributed to the conformational perturbance from covalent and non-covalent binding, which might make the enzyme structural rigidity. Increased accessibility to substrates of the enzymes displayed on the surface of the nano-granules might also benefit enzymatic catalysis [36, 37].

In addition, this study found that functional PHA granules could be easily separated to achieve a high recovery rate for repeat reactions. Only approximately 20% activity loss was observed after ten rounds of repeated use (Fig. 4f). The ability to recycle immobilized enzymes is economically desirable in many industrial processes. The reusability of engineered PHA granules depends on the characteristics of the target enzymes, and in most instances the activity of immobilized enzymes declines markedly after four rounds of repeated reactions [6, 34]. The high recovery of immobilized OPAA enzyme on PHA indicated a strong interaction between the protein and support material that prevented detachment and denaturation.

## Conclusions

This study provides a promising approach for one step in vivo immobilization of an organophosphorus hydrolase OPAA4301 on bacterial PHA nano-granules. Two display methods, mediated by PhaC or PhaP, were systematically examined and compared. Our results indicated that immobilization on PHA expanded the utility of OPAA4301. The tetrameric enzyme self-assembled and displayed on PHA granules with enhanced catalytic efficiency and stability, and reusability was excellent. Our results suggest that combination of covalent and non-covalent binding modes could be advantageous for display of other enzymes on PHA granules.

## Supplementary information

**Additional file 1.** Additional figures and tables.

**Additional file 2.** Protein groups.

## Data Availability

The datasets generated and analyzed during the current study are available from the corresponding author on reasonable request.
