# Supplementary material for: In vivo immobilization of an organophosphorus hydrolyzing enzyme on bacterial polyhydroxyalkanoate nano-granules
Source: Microb Cell Fact. 2019 Oct 10;18:166. doi: 10.1186/s12934-019-1201-2 (PMC6785862; doi:10.1186/s12934-019-1201-2)
Supplement: Supplementary file 1 — Additional file 1. Additional figures and tables. [file 12934_2019_1201_MOESM1_ESM.docx]

***In vivo* immobilization of an organophosphorus hydrolyzing enzyme on bacterial polyhydroxyalkanoate nano-granules**

Supplementary information

Ru Li ^1,2#^, Jian Yang ^1#^, Yunzhu Xiao ^1,3^, Lijuan Long ^1,2*^

^1^ CAS Key Laboratory of Tropical Marine Bio-Resources and Ecology, RNAM Center for Marine Microbiology, Guangdong Key Laboratory of Marine Materia Medica, South China Sea Institute of Oceanology, Chinese Academy of Sciences, Guangzhou 510301, People’s Republic of China

^2^ University of the Chinese Academy of Sciences, Beijing 100049, People’s Republic of China

^3^ Shenzhen Key Laboratory of Microbial Genetic Engineering, College of Life Sciences and Oceanology, Shenzhen University, Shenzhen, Guangdong, 518055, P. R. China

^#^These authors contributed equally to this work.

^*^Correspondence: longlj@scsio.ac.cn


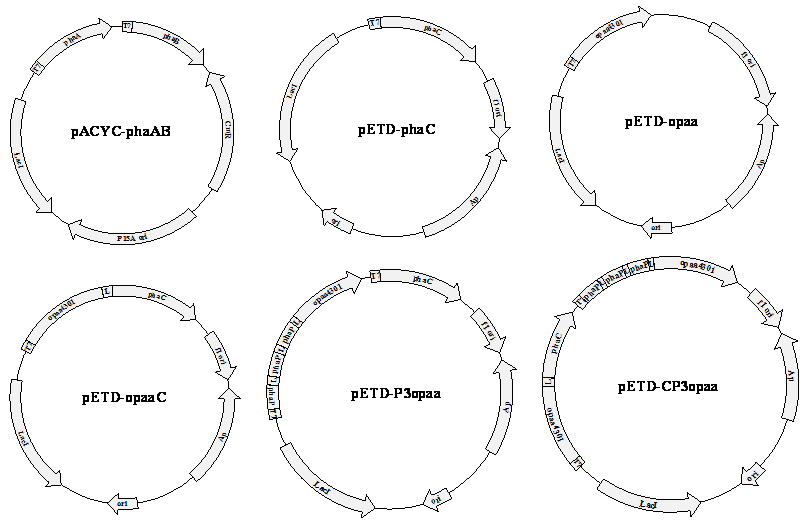
Figure S1 Expression vectors maps for *in vivo* production of immobilized of OPAA4301 on PHA


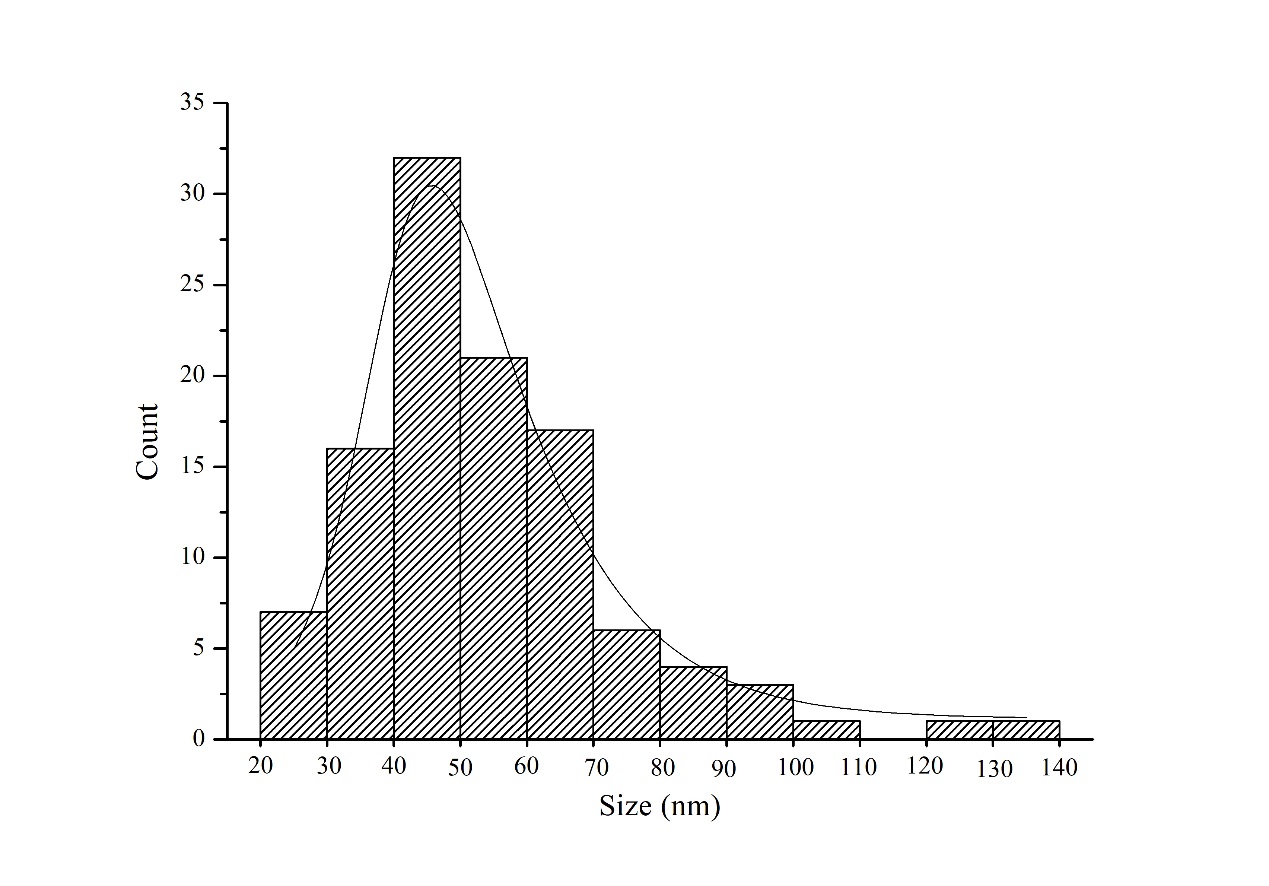


Figure S2 Particle size distribution of PHA produced by engineered *E. coli*. The analysis was carried out image pro plus based on TEM results.

**a**

**b**

Figure S3. The fourier-transform infrared (FT-IR) spectra of pure PHA granules (a) and CI-OPAA (b) extracted from *E.coli* cells.

**a**


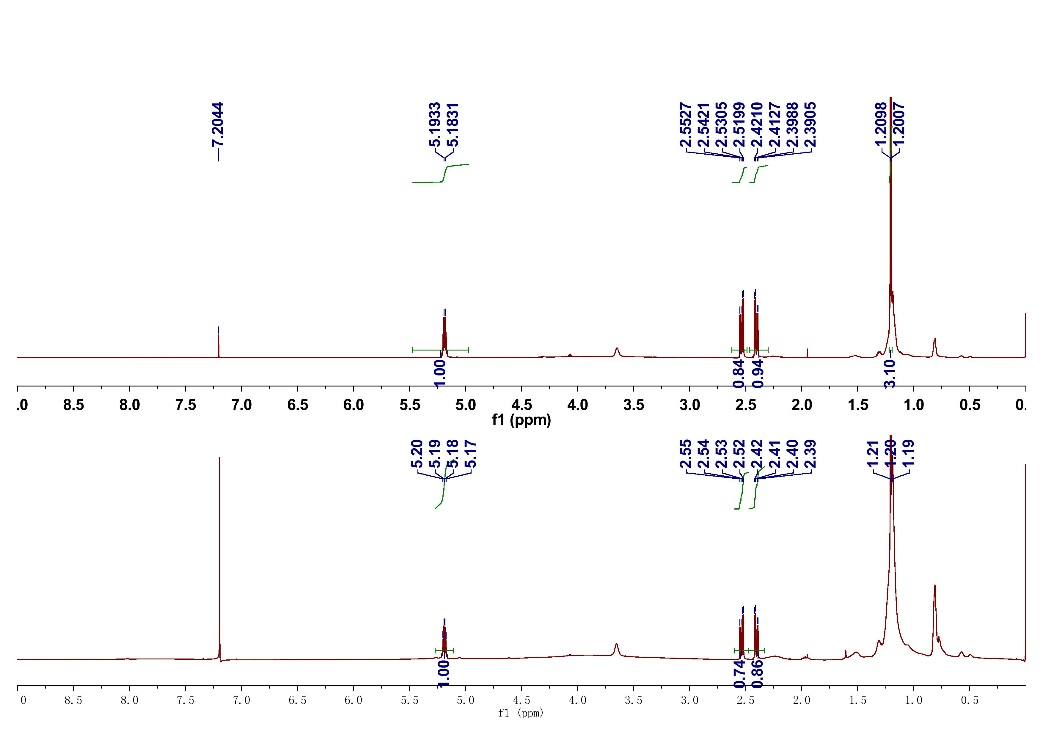


**b**


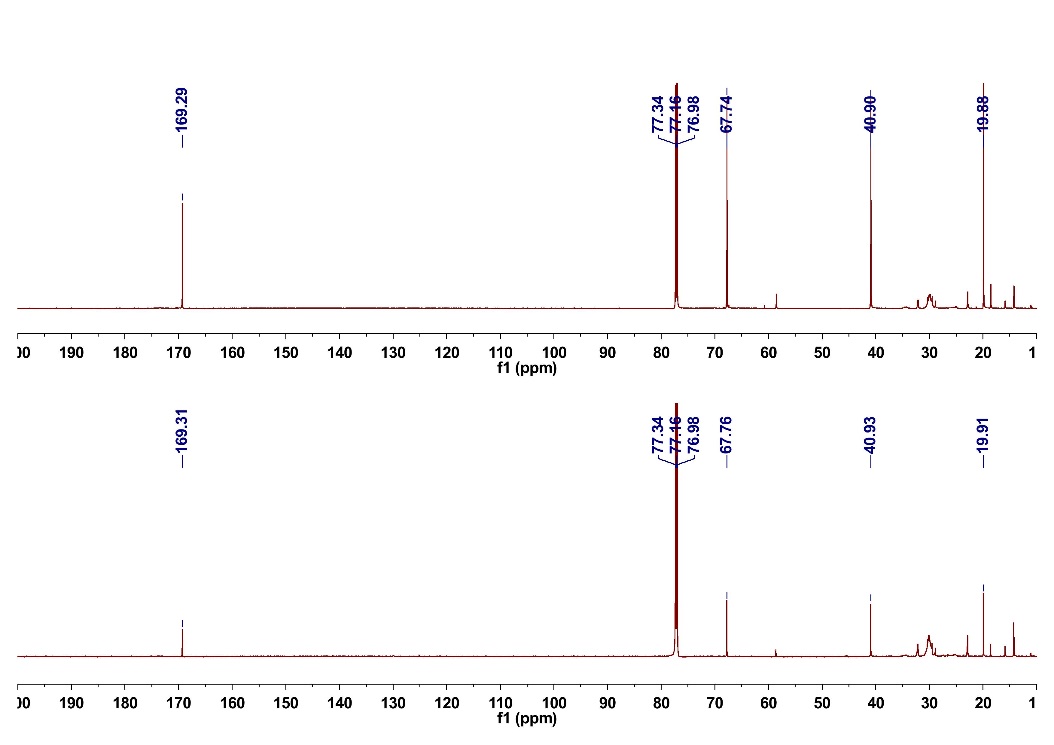


**c**


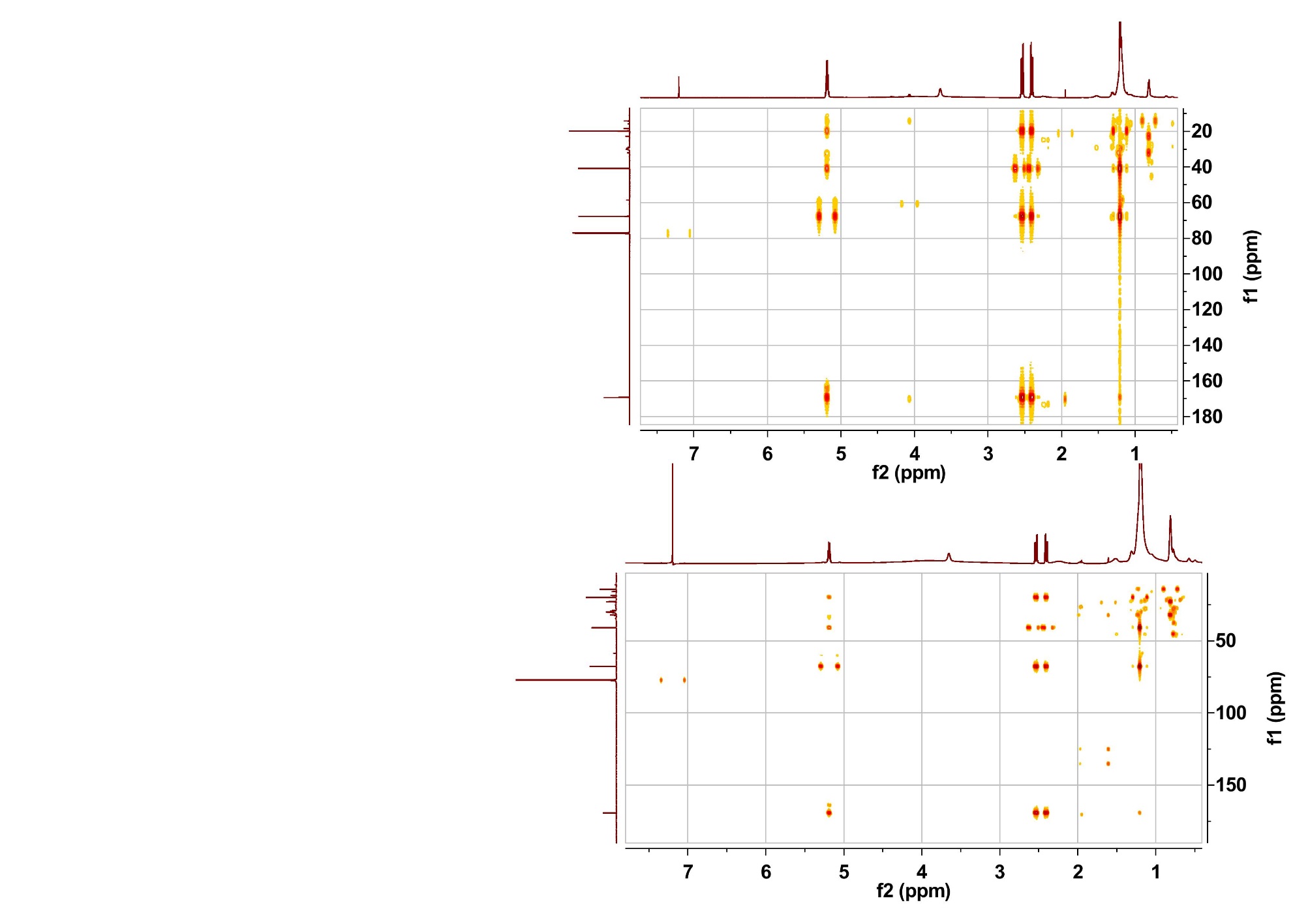


Figure S4 Nuclear magnetic resonance analysis of PHA nanoparticles. (a) ^1^H-NMR spectrum of PHA (uper) and CI-OPAA. (b) ^13^C-NMR spectrum of PHA (uper) and CI-OPAA. (c) HMBC spectrum of PHA (uper) and CI-OPAA. The ^1^H NMR data show four signals at *δ*_H_ 5.19 (1H, m, H-3), 2.54 (1H, dd, *J* = 15.5, 7.4 Hz, H-2a), 2.41 (1H, dd, *J* = 15.5, 5.8 Hz, H-2b) and 1.21 (3H, d, *J* = 8.7 Hz, H-4), suggesting existence of (3*R*)-hydroxybutanoic acid unit. The ^13^C NMR display four carbons attributable to one methyl (*δ*_C_ 19.9), one methylene (*δ*_C_ 40.9), one methine (*δ*_C_ 67.7), one ester carbonyl (*δ*_C_ 169.3), these NMR data disclose that compound consist of (3*R*)-hydroxybutanoic acid moiety, this conclusion further confirm by HMBC experiment.

Figure S5. The degradation of paraoxon by the immobilized organophosphorus hydrolase. The degradation experiments were carrying out according to previously reported work [1, 2]. Four sets of fresh cucumbers (320g) were contaminated with 50 μmol paraoxon for 30 min, then each contaminated cucumber was submerged in a solution (300ml) containing 5 mM glycine-NaOH (pH8.0) and 350 μg immobilized enzyme. The reaction mixture was incubated in room temperature, and aliquots of the washing solutions were withdrawn at different intervals of time and analyzed at 405 nm for the release of *p*-nitrophenol.

Table S1 Bacterial strains, plasmids, and oligonucleotides used in this study

| Strain name | Genotype, description, or sequence | Source/Refernces |
| --- | --- | --- |
| *E. coli* Trans-T1 | F^-^φ80(lacZ)ΔM15ΔlacX74hsdR(r_k_^-^,m_k_^+^)ΔrecA1398endA1tonA | TransGen Biotech |
| *E. coli* BL21(DE3) | F^−^ompThsdS_B_(r_B_^−^ m_B_^−^)galdcm (DE3) | TransGen Biotech |
| *Cupriavidus necator* | The PHA synthetic strain containing the gene phaA, phaB, phaP, phaC |  |
| Plasmids |  |  |
| pET-22b-opaa4301 | ampicillin resistance, pET-22b derivative containing the opaa4301 gene | Yunzhu Xiao[3] |
| pACYCDuet-1 | chloramphenicol resistance, dual T7 promoter | Novagen |
| pACYCD-phaAB | pACYCDuet-1 derivative containing the *phaA* gene under the first T7 promoter control and the *phaB* gene under the second T7 promoter | This study |
| pETDuet-1 | ampicillin resistance, dual T7 promoter | Novagen |
| pETD-opaaC | pETDuet-1 derivative containing the *phaC* gene, *linker* geng and *opaa4301* gene under the control of the first T7 promoter | This study |
| pETD-P3opaa | pETDuet-1 derivative containing the *phaC* under the first T7 promoter control and the three *phaP* gene, *linker* gene and *opaa4301* gene under the second T7 promoter control | This study |
| pETD-CP3opaa | pETDuet-1 derivative containing the *opaa4301-l-phaC* gene under the first T7 promoter control and the *phaP×3-l-opaa4301* gene under the second T7 promoter | This study |
| Oligonucleotides |  |  |
| phaA no start Nco I | 5’-AGATATACCATGGGCACTGACGTTGTCATCGTATC-3’ | This study |
| phaA no stop Not IⅠ | 5’-GCATTATGCGGCCGCTTTGCGCTCGACTGCCAGCGCCAC-3’ | This study |
| phaB no start Nde I | 5’-GGAGATATACATATGACTCAGCGCATTGCGTATGTGACC -3’ | This study |
| phaB stop Kpn I | 5’-AGACTCGAGGGTACCTTAGCCCATATGCAGGCCGCCGTTGA-3’ | This study |
| Linker-1 | GGTGGTGGTAGCGGTGGTGGTTCTGGTGGTGGTAGC | GanQiao Ran[4] |
| Linker-2 | AACAATAACAACAACCTCGGGATCGAGGGAAGGATTTCA | Banki, M. R.[5] |
| Opaa4301 no start Nco I | 5’-AGATATACCATGGGCGATAAATTAGCGGTGTTATATG-3’ | This study |
| Opaa4301-L1 | 5’-CAGAACCACCACCGCTACCACCACCATCTAAGTGTAGATCACGGG-3’ | This study |
| PhaC-L1 | 5’-GGTGGTGGTAGCGGTGGTGGTTCTGGTGGTGGTAGCGCGACCGGCAAAGGCGCGGCAGCTTCCACGCAG-3’ | This study |
| phaC stop Not I | 5’-GCATTATGCGGCCGCTCATGCCTTGGCTTTGACGTATCGCCCAGGCGCG-3’ | This study |
| phaC no start Nco I | 5’-AGATATACCATGGGCGCGACCGGCAAAGGCGCGGCAGCTTCCACGCAG-3’ | This study |
| phaC no stop Not I | 5’-GCATTATGCGGCCGCTGCCTTGGCTTTGACGTATCGCCCAGGCGCG-3’ | This study |
| phaP Nde I | 5’-GCGCCATATGATCCTCACCCCGGAACAAGTTGCAGC-3’ | This study |
| phaP-L2 Bgl II | 5’-GCGC AGATCTTGAAATCCTTCCCTCGATCCCGAGGTTGTTGTTATTGTTGGCAGCCGTCGTCTTCTTTGCCGT-3’ | This study |
| phaP Bgl II | 5’-GCGCAGATCTATCCTCACCCCGGAACAAGTTGC-3’ | This study |
| phaP-L2 Kpn I | 5’-GCGC GGTACCTGAAATCCTTCCCTCGATCCCGAGGTTGTTGTTATTGTTGGCAGCCGTCGTCTTCTTTGCCGT-3’ | This study |
| phaP Kpn I | 5’-GCGCGGTACCATCCTCACCCCGGAACAAGTTGC-3’ | This study |
| phaP-L2 | 5’-TGAAATCCTTCCCTCGATCCCGAGGTTGTTGTTATTGTTGGCAGCCGTCGTCTTCTTTGCCGT-3’ | This study |
| Opaa4301-L2 | 5’-GAGGGAAGGATTTCAGATAAATTAGCGGTGTTATATGCCG-3’ | This study |
| Opaa4301 stop Avr II | 5’-GCGCCCTAGGTCAATCTAAGTGTAGATCACGGG-3’ | This study |
| Opaa4301 EcoR I | 5’-GCGCGAATTCGGATAAATTAGCGGTGTTATATG-3’ | This study |
| phaC no stop Afl II | 5’-GCGCCTTAAGTCATGCCTTGGCTTTGACGTATC-3’ | This study |
| phaC no start Nco I | 5’-AGATATACCATGGGCGCGACCGGCAAAGGCGCGGCAGCTTCCACGCAG-3’ | This study |
| phaC stop Not I | 5’-GCATTATGCGGCCGCTCATGCCTTGGCTTTGACGTATCGCCCAGGCGCG-3’ | This study |

**References:**

1. Del GI, Coppolecchia R, Merone L, Porzio E, Carusone TM, Mandrich L, Worek F, Manco G: An efficient thermostable organophosphate hydrolase and its application in pesticide decontamination**.** Biotech Bioeng. 2016, 113**:**724-734.

2. Bai YP, Luo XJ, Zhao YL, Li CX, Xu DS, Xu JH: Efficient degradation of malathion in the presence of detergents using an engineered organophosphorus hydrolase highly expressed by Pichia pastoris without methanol induction**.** J Agr Food Chem. 2017, 65**:**9094.

3. Xiao Y, Yang J, Tian X, Wang X, Li J, Zhang S, Long L: Biochemical basis for hydrolysis of organophosphorus by a marine bacterial prolidase**.** Process Biochem. 2016, 52.

4. Ran G, Tan D, Dai W, Zhu X, Zhao J, Ma Q, Lu X: Immobilization of alkaline polygalacturonate lyase from Bacillus subtilis on the surface of bacterial polyhydroxyalkanoate nano-granules**.** Appl Microbiol Biotechnol. 2017, 101**:**3247-3258.

5. Banki MR, Gerngross TU, Wood DW: Novel and economical purification of recombinant proteins: intein-mediated protein purification using in vivo polyhydroxybutyrate (PHB) matrix association**.** Pro Sci. 2005, 14**:**1387-1395.
